# Reactive Countermovement Jump Mechanics Across Basketball Experience Levels

**DOI:** 10.3390/jfmk11040406

**Published:** 2026-10-04

**Authors:** Branislav Božović, Jelena Aleksić, Radivoj Mandić, Miloš Mudrić, Olivera M. Knežević, Dragan M. Mirkov

**Affiliations:** Faculty of Sport and Physical Education, University of Belgrade, 11030 Belgrade, Serbia; branislav.bozovic.91@gmail.com (B.B.); jelena.aleksic@fsfv.bg.ac.rs (J.A.); radivoj.mandic@fsfv.bg.ac.rs (R.M.); milos.mudric@fsfv.bg.ac.rs (M.M.); olivera.knezevic@fsfv.bg.ac.rs (O.M.K.)

**Keywords:** countermovement jump, rapid initiation, movement time, force–time strategy, basketball, motor control, arm swing

## Abstract

Countermovement jumps are usually tested as self-initiated maximal efforts, although many sport actions require athletes to react to an external event and still jump as high as possible. **Background and Objectives:** We examined how this rapid-initiation requirement was associated with CMJ mechanics, whether the pattern was similar across levels of basketball-specific experience, and whether arm swing altered the response. **Methods:** Forty-four men (15 physically active non-basketball controls, 15 mid-level basketball players, and 14 high-level basketball players) performed countermovement jumps with and without arm swing in four fixed-order blocks. Trial-level linear mixed-effects models included 564 valid attempts. **Results:** In the signal-initiated blocks, movement time was 0.091 s shorter (95% CI −0.103 to −0.078) and jump height was 1.63 cm lower (95% CI −2.04 to −1.21). Unloading (−0.069 s), braking (−0.011 s), and propulsion (−0.010 s) were all shorter; countermovement depth decreased, peak downward speed increased, and net propulsive impulse fell by 0.052 N s/kg despite a small increase in mean net propulsive force. Movement time decreased in all six group-by-jump cells. The jump-height reduction was statistically clear at the group level but small and of uncertain practical relevance for individuals. Basketball-specific experience did not clearly moderate the main condition effects. Arm swing reduced the movement-time difference by 0.043 s but did not clearly reduce the jump-height difference. **Conclusion:** The rapid-initiation condition was associated with a broad reorganization of movement timing and force–time strategy, with a smaller change in endpoint performance. Because the conditions were always performed in the same order, these findings should be interpreted as fixed-order condition differences and confirmed in counterbalanced designs.

## 1. Introduction

The countermovement jump (CMJ) is widely used to assess lower-body performance and neuromuscular status. Jump height is easy to interpret, but it says little about how the result was achieved. Force–time analysis makes that strategy visible by separating unloading, braking, and propulsion [1,2]. This distinction matters because similar jump heights can arise from different combinations of movement time, countermovement depth, force, and impulse [3,4,5].

Earlier work has shown that the mechanics of a maximal vertical jump can change much more than the final jump height. Mandic, Jakovljevic, and Jaric varied countermovement depth over a wide range in professional basketball players and found large changes in force and power output but relatively small changes in maximal-jump height [6]. A later study of elite basketball players and physically active non-athletes showed that the preferred countermovement depth was consistently shallower than the depth that maximized jump height [7]. This suggests that the control of a maximal jump is not organized around jump height alone. Training studies point in the same direction. Markovic and colleagues reported similar improvements in jump height after training with different external loads, despite load-specific adaptations in CMJ depth, duration, force, and power [8]. Taken together, these studies show that similar endpoint performance can be achieved through different mechanical solutions.

Arm swing adds another source of flexibility. In self-initiated CMJs, it generally increases jump height and changes the force–time pattern through its effects on segmental coordination, movement duration, and propulsive impulse [9,10,11,12,13]. These additional degrees of freedom may become especially important when the jump must be initiated and completed quickly.

Most laboratory CMJ tests are self-paced: participants choose when to start and are instructed mainly to jump as high as possible [14,15]. Many sport actions pose a different problem because an external event determines when the movement should begin, making rapid initiation part of the task itself. This is not the same as visual distraction or a conventional dual-task paradigm; the signal tells the athlete when to start the primary motor action. When CMJs are performed for quickness, shorter time to take-off has been linked to shorter movement phases, a shallower countermovement, and changes in force and impulse [16,17,18]. Likewise, stimulus-triggered CMJ research combining force-platform and electromyographic measurements has shown that the stimulus can alter reaction and early unweighting timing without a proportional change in final jump performance [19,20,21].

The task used here combines two demands within the same movement: start as quickly as possible after the go-signal and still jump as high as possible. This shifts the question from whether the signal changes jump height to how performers solve the added time constraint. Based on earlier work on countermovement depth and load-specific adaptation [6,7,8], we expected any adjustment to be visible across the force–time sequence—in preparation time, countermovement depth and downward velocity, force production, the time available to generate impulse, or some combination of these features.

Basketball frequently requires players to initiate jumps in response to changing visual information while minimizing the time available for movement preparation. Repeated exposure to these demands may influence how basketball players organize countermovement depth, phase durations, force production, and impulse when rapid initiation is required [22,23,24]. Previous research has shown differences between elite basketball players and physically active non-athletes in preferred countermovement strategy [7], while some CMJ force–time characteristics may also differ across competitive levels [23]. However, expertise-related advantages in reaction performance depend strongly on the specificity of the stimulus-response relationship [25,26,27,28,29]. Because the simple color-change signal used in the present study contained little basketball-specific information, basketball experience was not necessarily expected to shorten reaction time directly. Instead, experience was expected to modify the magnitude or mechanical expression of the difference between the two task conditions.

Accordingly, our primary aim was to determine how the mechanical organization of a maximal CMJ differs when participants are required to initiate movement rapidly after a visual go-signal compared with self-initiated jumping. We also examined whether the pattern was consistent across groups with different levels of basketball-specific experience and whether arm swing changed the response. Because the visually triggered rapid-initiation condition was always performed after the self-initiated condition, this comparison was intended to characterize performance under the two task conditions rather than to isolate the independent effect of the visual stimulus from possible order-related effects. We hypothesized that (H1) the rapid-initiation condition would be accompanied by coordinated temporal, kinematic, and kinetic changes rather than by an isolated change in jump height; (H2) basketball-specific experience would modify the magnitude and/or mechanical expression of this response, although the mixed prior evidence did not justify a simple linear expertise gradient or a directional prediction for individual variables; and (H3) arm swing would alter the mechanical solution used under the added time constraint.

## 2. Materials and Methods

### 2.1. Study Design

We used a repeated-measures laboratory design with jump type and initiation condition as within-participant factors. The two jump types were the countermovement jump without arm swing (CMJ) and the countermovement jump with arm swing (CMJ-A). Each was performed as a self-initiated jump and as a signal-initiated jump, in which participants were instructed to begin as quickly as possible after a visual go-signal while still jumping maximally. Participant group was the between-participant factor, with three categories: physically active non-basketball controls, mid-level basketball players, and high-level basketball players. All testing was completed in a single session. Because every participant performed the four blocks in the same order, initiation condition is confounded with block order; the analyses therefore quantify fixed-order condition differences rather than the isolated causal effect of the go-signal.

### 2.2. Participants

The study included 44 men allocated to three groups according to basketball participation and current competitive level. The non-basketball group (i.e., control group) comprised 15 physically active participants with no organized basketball experience (age 24.3 ± 1.9 years, stature 183.5 ± 5.4 cm, body mass 76.7 ± 6.6 kg). These participants were students at the Faculty of Sport and Physical Education and were physically active at the time of testing. The mid-level basketball group comprised 15 players with at least five years of basketball training and competitive experience (age 24.1 ± 3.4 years, stature 187.1 ± 6.4 cm, body mass 82.4 ± 8.5 kg). At the time of testing, they competed in the second or third tier of Serbian basketball, but not in the highest domestic leagues. The high-level basketball group included 14 players with at least two years of experience at the highest competitive levels represented in the sample—the Adriatic Basketball Association League (ABA League) or the Basketball League of Serbia (age 22.4 ± 2.4 years, stature 195.6 ± 6.4 cm, body mass 88.3 ± 8.0 kg). Players in both basketball groups (mid-level and high-level) completed at least five team practices per week. Session duration, total weekly training volume, resistance or plyometric training exposure, competition frequency, and recent training history were not systematically recorded. Similarly, although the non-basketball participants were physically active students at the Faculty of Sport and Physical Education, their weekly physical activity and training volume were not quantified. Accordingly, the groups were classified according to basketball participation and current competitive level rather than detailed measures of current training exposure. Participants had no musculoskeletal injury at the time of testing and provided written informed consent before participation. The study followed the Declaration of Helsinki and was approved by the Ethics Committee of the Faculty of Sport and Physical Education, University of Belgrade (protocol 02-3042/23-1, 18 December 2023). An a priori sample-size calculation was conducted in G*Power (version 3.1.9.7) using a repeated-measures ANOVA with a within–between interaction as an approximation for the planned primary Group × Initiation Condition analysis. Assuming *f* = 0.25, α = 0.05, 90% power, three groups, two initiation conditions, a repeated-measures correlation of 0.60, and ε = 1, the required sample was 45 participants. The final sample included 44 participants. This calculation did not specifically address higher-order interactions or the trial-level mixed-effects model.

### 2.3. Experimental Procedure

Participants completed a standard warm-up consisting of 5 min of stationary cycling at a self-selected pace, followed by calf raises, hip hinges, lunges, squats, and hopping. They then performed one or two familiarization jumps in each of the four conditions and rested for 2 min before the main test.

The four blocks were completed in the following fixed order: (1) self-initiated CMJ without arm swing, (2) signal-initiated CMJ without arm swing, (3) self-initiated CMJ-A, and (4) signal-initiated CMJ-A. The order was not randomized or counterbalanced; therefore, within each jump type, the signal-initiated block always followed the corresponding self-initiated block. This within-jump-type sequence was selected so that the self-initiated block preceded the corresponding rapid-initiation block, with the intention of reducing carryover of the rapid-initiation instruction into recorded self-initiated performance. However, this rationale did not eliminate potential carryover between jump types or other effects associated with the fixed block order. The protocol aimed to obtain five acceptable attempts per block. Attempts that were clearly invalid during data collection because of improper landing, prolonged marker occlusion or detachment, or failure to respond within the acceptable interval were repeated when possible. Testing within each block ended after five attempts had been judged acceptable during data collection. During subsequent offline processing, trials that did not satisfy the final data-quality and inclusion criteria were excluded and could not be replaced, resulting in between one and five valid attempts per participant-by-jump-by-condition cell.

In all four conditions, participants were instructed to jump as high as possible and land approximately in the same location. For the self-initiated CMJ trials, participants stood on the force plates with their hands on their hips. For self-initiated CMJ-A trials, participants stood with their arms extended approximately 20–30° in front of the body. Although this standardized starting position may have influenced movement initiation, it was necessary to prevent occlusion of the markers placed over the hips. In the self-initiated conditions, participants remained stationary for 5 s before initiating the jump at their own discretion.

In the signal-initiated trials, the research assistant indicated that the trial was ready, after which participants watched the screen and initiated the jump as quickly as possible following the visual go-signal while still attempting to jump maximally. The visual go-signal appeared after a randomized delay of 3–10-s. The additional procedures and instructions used in the signal-initiated conditions are described in Section 2.4.

The rest interval between trials in each block was 30–60 s, with longer intervals used during the signal-initiated blocks, and the rest interval between blocks was 2 min. Attempts were excluded and repeated when participants did not land properly, occluded or detached a marker for a prolonged period, or failed to respond to the visual signal within the acceptable interval, as determined by the research assistant during data collection. Participants were instructed to avoid strenuous training and competition during the 24-h period preceding testing, and compliance with this requirement was confirmed by the research assistant before testing. Testing was conducted within a standardized time-of-day period, and all participants wore athletic footwear (sneakers). All testing was completed in a single session using the same standardized warm-up and testing procedures.

### 2.4. Visual Go-Signal and Rapid-Initiation Task

The visual go-signal was presented with a custom LabVIEW application (LabVIEW 2012, National Instruments, Austin, TX, USA) on a screen positioned 2 m in front of the participant at eye level. The display operated at a refresh rate of 60 Hz, as standard by the manufacturer. The screen displayed a black circle on a gray background. At a random time within a 10-s period, the circle changed from black to white, but it never appeared before the third second. In addition to the maximal-jump instruction that was given in all jump conditions, in signal-initiated trials participants were instructed to initiate the jump as quickly as possible when the circle changed color. Thus, the signal-initiated condition combined a visual go-signal with an explicit rapid-initiation requirement.

To discourage anticipatory movement initiation, catch trials were randomly interspersed among the signal-initiated trials. During a catch trial, the circle did not change color and the participant was required to withhold the jump. One catch trial was randomly inserted within every 15 signal presentations. After a catch trial, the participant returned to the standardized starting position and prepared for the next attempt. Catch trials were not included in the analysis. The randomized 3–10 s delay and catch trials were used to reduce temporal prediction and premature movement initiation. Reaction time was defined as the interval from the visual go-signal to the first detectable change in vertical ground-reaction force marking movement onset.

### 2.5. Instrumentation

Vertical ground-reaction force was recorded at 1000 Hz with two fixed three-dimensional AMTI BP600400 force platforms (AMTI, Watertown, MA, USA), one under each foot, as described for the same laboratory setup [30]. The left and right vertical force signals were smoothed separately with the MATLAB smooth function, summed to obtain total vertical force, and smoothed again. The platforms were calibrated according to the manufacturer’s instructions, and force data were exported through the AMTI acquisition application. An AMTI MSA-6 Mini Amp strain-gauge amplifier was used along with the AMTI Net Force software (Version 2.4.0).

Kinematic data were recorded at 300 Hz with four Qualisys Miqus M3 infrared cameras (Qualisys Medical AB, Gothenburg, Sweden). The cameras were positioned on the left side of the body, with infrared markers placed on shoulder, hip, knee, lateral malleolus, heel and the head of the fifth metatarsal bone. The heel and metatarsal markers were placed over the shoes on places closest to referent point. The *X*-axis was directed from posterior to anterior and the *Z*-axis upward. The system origin was aligned with the floor point below the standing center-of-mass, and participants placed their feet at the marked origin. The camera system was calibrated before recording. The system was calibrated before each testing session with the volume set at the 4 × 4 × 4 m in order to cover the full range of potential motion. The 300.6 mm wand was used for system calibration with the average residual calibration error less than 1 mm. Marker trajectories were processed in Qualisys Track Manager 2020.3 and exported as TSV files. Missing markers were interpolated in QTM, and a moving-average smoother was used when needed. The camera and marker setup has been described in detail elsewhere [31].

### 2.6. Signal Processing and Event Definitions

Biomechanical variables were calculated with custom MATLAB scripts (R2023b, MathWorks, Natick, MA, USA). Marker coordinates were converted from millimeters to meters. Vertical center-of-mass and toe velocities were calculated by first differences and smoothed with the Savitzky–Golay method using a 20-sample window. The processed outputs included phase durations, force–time variables, center-of-mass movement, jump-height estimates, reaction time, and sagittal-plane joint angles.

For force processing, a preliminary body-weight value was calculated from samples 100 to 3000 (approximately 0.10–3.00 s). Samples from approximately 0.10 to 3.50 s that were within one standard deviation of this preliminary mean were retained to calculate body weight. Body mass was body weight divided by 9.81 m/s^2^, and net vertical force was total vertical force minus body weight. A later segment (approximately 6.50–7.95 s) was used as a drift check, but no automatic drift correction was applied. A second-order, 50 Hz low-pass Butterworth filter with zero-phase filter processing was used for differentiated force signals in the rate-of-force-development calculations. Acceleration was net force divided by body mass; velocity and displacement were calculated by cumulative trapezoidal integration from movement onset.

Movement onset was identified from net vertical force before the unweighting minimum. The threshold was the pre-movement mean minus five standard deviations, and the signal was searched backward from the force minimum to locate the boundary crossing. The script also calculated an RFD-based onset candidate but used the force-threshold onset when it was available. Take-off and landing were the first and last samples, respectively, in the flight segment where net force was within 20 N of the minimum flight value. The visual stimulus and force-platform recording were synchronized using a trigger signal generated by a custom-made LabVIEW program (National Instruments LabVIEW 2012, Austin, TX, USA). The trigger provided a common temporal reference for the onset of the visual stimulus and force-platform data acquisition.

The end of unloading was the minimum vertical center-of-mass velocity. The end of braking was the minimum center-of-mass displacement, where vertical velocity changed from downward to upward. Propulsion lasted from this lowest position to take-off. Movement time was the sum of unloading, braking, and propulsion durations. Net impulse from movement onset to take-off was divided by body mass to obtain take-off velocity. Impulse-momentum jump height was calculated as take-off velocity squared divided by 2 g and was the main jump-height outcome. Jump height from flight time was kept as a secondary measure.

RSImod was recalculated for every attempt as impulse-momentum jump height in meters divided by movement time in seconds. Impulse-momentum jump height was the same height outcome used in the main analysis and was calculated from take-off velocity as v^2^/(2 g). Movement time was the interval from movement onset to take-off and was calculated as unloading time plus braking time plus propulsion time. Therefore, RSImod = impulse-momentum jump height/(unloading time + braking time + propulsion time) [32,33].

Sagittal-plane joint angles were calculated from the left-side marker coordinates. The ankle angle was defined with fifth metatarsal bone-lateral malleolus–knee marker segments, the knee angle using the lateral malleolus–knee–hip marker segments, and the hip angle using the knee–hip–shoulder marker segments. The reported values represented the angles corresponding to the lowest center-of-mass position. Angles were expressed in degrees, with the inner angle calculated such that larger values indicated less flexion. The reported event corresponded to the point of maximal countermovement depth, defined as the transition from the braking to the propulsive phase.

### 2.7. Outcomes and Normalization

Table 1 contains the main outcomes grouped according to the question they addressed. Jump height represented endpoint performance, whereas movement time described the interval from movement onset to take-off. RSImod was retained as a combined height–time measure and interpreted together with both of its components. Reaction time and total signal-to-take-off time were available only for signal-initiated trials. Changes in jump mechanics were characterized using propulsive impulse and its force–time components, preparation-phase durations, countermovement depth, peak downward speed, and selected sagittal-plane joint angles.

Force and impulse variables were divided by body mass and reported as N/kg and N s/kg. Countermovement depth was divided by body stature. Time, velocity, and joint angles were not normalized to body mass. Mass normalization reduces the direct effect of body mass, but it does not remove all differences in body size or segment length between groups. Other jump-height estimates, take-off velocity, power, rate of force development, additional impulse variables, leg stiffness, and landing variables were not treated as separate primary outcomes.

### 2.8. Trial Inclusion and Analysis Dataset

Although the protocol aimed to obtain five acceptable attempts per block, trials that did not satisfy the final data-quality and inclusion criteria were excluded during offline processing and could not be replaced. The processed dataset contained 568 rows. Four administrative rows with participant identifiers of zero or less were removed, leaving 564 recorded attempts from 44 participants. No duplicate participant-by-jump-by-condition-by-trial records were present. The dataset comprised 176 participant-by-jump-by-condition cells, with one to five valid attempts per cell. All 564 attempts had complete data for the three principal outcomes, and all 284 signal-initiated attempts had valid reaction times between 0 and 1 s.

All retained attempts were included in the main mixed-effects analyses; attempts were not selected on the basis of jump height. Participant means were calculated only for the descriptive tables, providing one descriptive value per participant in each cell. To determine whether the findings depended on trial selection, the main models were repeated using up to three first valid attempts and, separately, up to three highest valid jumps in each cell.

### 2.9. Statistical Analysis

All analyses were independently reproduced in Python 3.12.13 using NumPy 2.3.5 and Pandas 3.0.1. Linear mixed-effects models were fitted by restricted maximum likelihood with a generalized least-squares implementation and a participant random intercept, reflecting the repeated attempts contributed by each participant. The reproduced analysis agreed with the archived output across 321 estimates, standard errors, confidence intervals, and *p*-values. Model checks covered convergence, intraclass correlation, residual skewness and kurtosis, residual-versus-fitted patterns, condition-specific residual variation, and extreme standardized residuals. Participant-clustered sandwich standard errors were used as a robustness check for the three principal condition contrasts. Because initiation condition was manipulated within participants, the three principal outcome models were also refitted with a participant-specific random slope for initiation condition to allow the size of the condition effect to vary across individuals.

Models for jump height, movement time, RSImod, propulsive variables, preparation variables, and selected joint angles included initiation condition, jump type, two orthogonal group contrasts, and all corresponding two-way and three-way interactions. Trial number was centered within each participant, jump type, and condition block to account for a linear practice or fatigue trend within blocks. The first group contrast compared both basketball groups with controls (weights −2/3, 1/3, and 1/3 for control, mid-level, and high-level), representing basketball-specific experience. The second compared high-level with mid-level players (weights 0, −1/2, and 1/2), representing the competitive level within basketball. Jump type and condition were effect-coded as −1/2 and 1/2.

The statistical tests followed the three hypotheses stated above. H1 was evaluated from the overall condition contrasts across the performance, preparation, and propulsion outcomes, with emphasis on whether related variables changed as a coherent mechanical pattern. H2 was evaluated from group-by-condition interactions and, where relevant, group-by-jump-type-by-condition interactions. H3 was evaluated primarily from jump-type-by-condition interactions. Higher-order interactions and isolated secondary effects outside these questions were treated as exploratory.

The Holm correction was applied separately to the three principal performance/timing outcomes (jump height, movement time, and RSImod) and to the four preparation outcomes (unloading time, braking time, countermovement depth relative to stature, and peak downward speed). Propulsive net impulse was the principal propulsion outcome; mean net propulsive force and propulsion duration were used to explain its mechanical basis. Joint-angle outcomes were secondary. Reaction time and signal-to-take-off time were analyzed only in signal-initiated attempts using the same group contrasts, jump type, centered trial number, and a participant random intercept. Associations between reaction time and jump height were separated into within-participant and between-participant components.

Model-based contrasts are reported with 95% confidence intervals and exact two-sided *p*-values. The main model tests and confidence intervals used 41 participant-level degrees of freedom; participant-clustered sensitivity tests used 43 degrees of freedom. Statistical significance was set at alpha = 0.05. Non-significant interactions were taken to indicate a lack of clear evidence for moderation, not equivalence between groups. Descriptive results are presented as participant-level mean ± standard deviation for each cell.

### 2.10. AI-Assisted Support and Author Oversight

During manuscript preparation, the authors used Claude (Anthropic) and Codex (OpenAI) to assist with statistical code checking and preparation of tables and figures. These tools were not used for participant testing or data collection. All AI-assisted outputs, numerical results, tables and figures were reviewed and verified by the authors against the study data and archived analyses. The authors take full responsibility for the content, accuracy, and originality of the manuscript.

## 3. Results

### 3.1. Analysis Sample

The analysis included 564 valid attempts from 44 participants: 15 controls, 15 mid-level basketball players, and 14 high-level basketball players. Each participant-by-jump-by-condition cell contained between one and five valid attempts. Data for jump height, movement time, and RSImod were available for every attempt. The signal-initiated blocks contributed 284 attempts, all with a valid reaction-time values between 0 and 1 s.

### 3.2. Mechanical Response to the Rapid-Initiation Requirement

**Figure 1:**
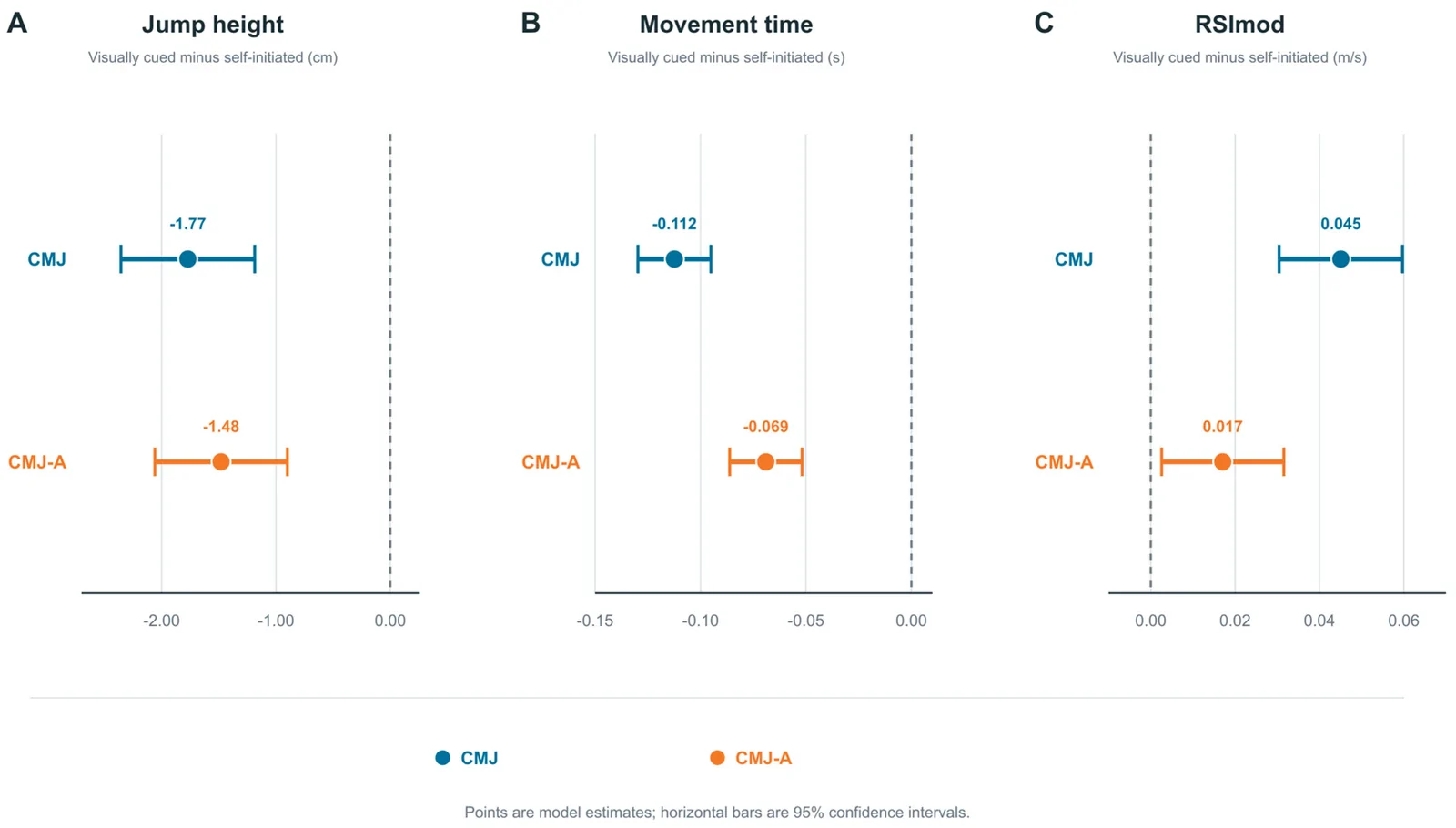
Model-estimated differences between the signal-initiated and self-initiated blocks for CMJ and CMJ-A. Estimates are averaged across the planned group contrasts. Points show the estimated difference, and horizontal bars show 95% confidence intervals. Negative values indicate lower jump height or shorter movement time in the signal-initiated block; positive values indicate higher RSImod. Because block order was fixed, the estimates describe condition differences rather than an isolated causal effect of the visual signal.

Table 2 summarizes the principal outcomes, and Figure 1 shows the model-estimated condition differences by jump type. Averaged across groups and jump types, movement time was 0.091 s shorter in the signal-initiated blocks (95% CI −0.103 to −0.078, *p* < 0.001), while jump height was 1.63 cm lower (95% CI −2.04 to −1.21, *p* < 0.001). RSImod increased by 0.032 m/s (95% CI 0.023 to 0.041, *p* < 0.001) because movement time decreased proportionally more than jump height. All three effects remained significant after Holm correction. The timing response was especially consistent: movement time decreased in all six group-by-jump cells, with model-estimated differences from −0.045 to −0.124 s. Jump height also decreased in every cell, but less uniformly (−0.45 to −2.81 cm). The clearest response to the rapid-initiation condition was therefore a change in how the movement was organized, rather than a uniform loss of jump height.

### 3.3. Force–Time Reorganization

**Figure 2:**
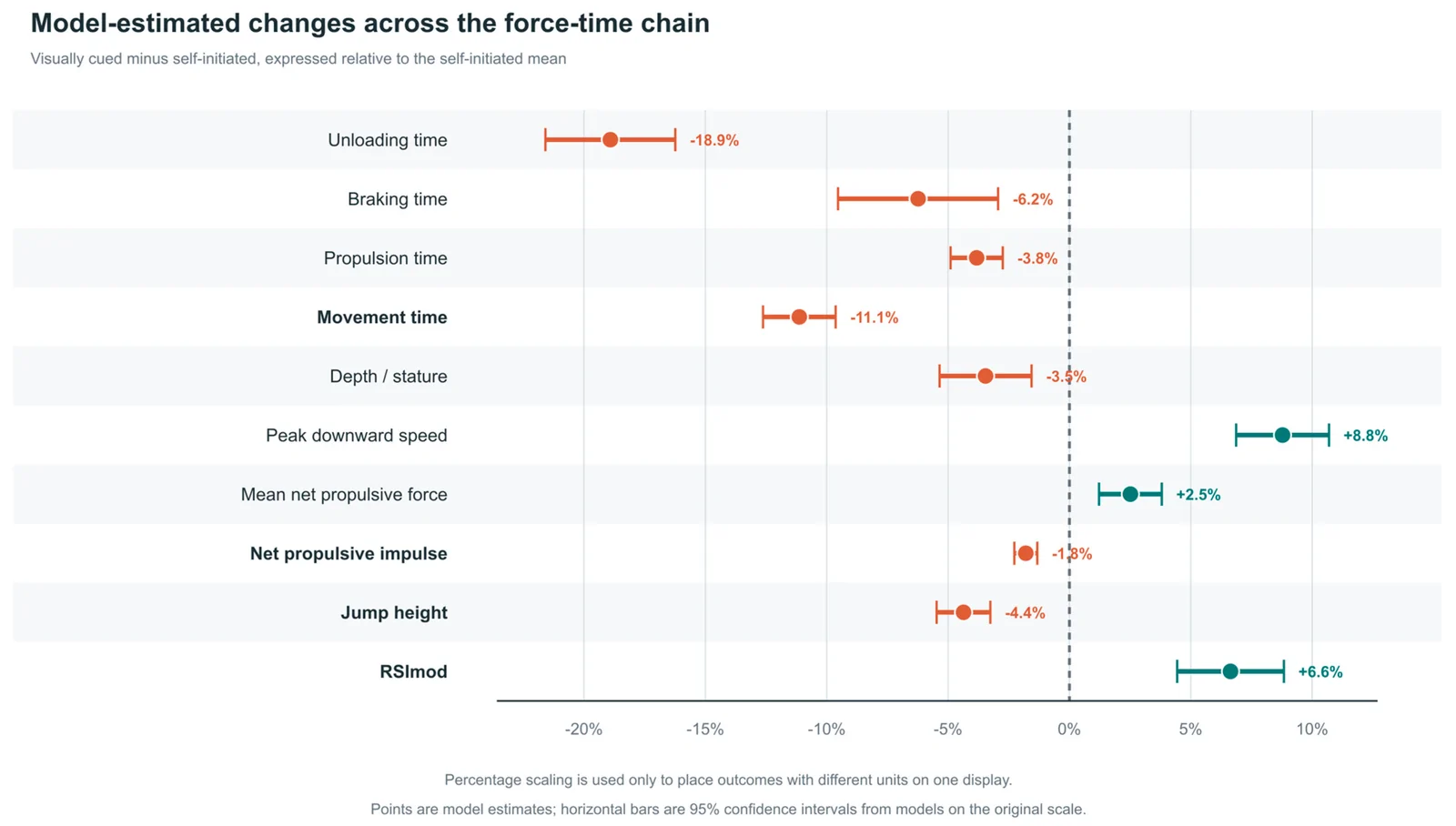
Model-estimated changes across the force–time chain under the rapid-initiation requirement. To place outcomes with different units on one display, each estimate and confidence interval are expressed as a percentage of the corresponding self-initiated participant mean. The mixed models were fitted on the original measurement scales. Points show estimates, and horizontal bars show 95% confidence intervals.

The shorter movement time reflected changes across the force–time sequence (Figure 2). Body-mass-normalized net propulsive impulse was lower in the signal-initiated blocks (−0.052 N s/kg, 95% CI −0.066 to −0.039, *p* < 0.001). Mean net propulsive force was slightly higher (+0.274 N/kg, 95% CI 0.133 to 0.415, *p* < 0.001), whereas propulsion duration was 0.010 s shorter (95% CI −0.013 to −0.008, *p* < 0.001). In other words, participants did not produce less mean propulsive force; they applied force for less time, and the small increase in force was not enough to preserve propulsive impulse.

Table 3 shows that the adjustment began before propulsion. Unloading duration decreased by 0.069 s and braking duration by 0.011 s. Countermovement depth relative to stature decreased by 0.0058, while peak downward speed increased by 0.103 m/s; all four preparation outcomes remained significant after Holm correction. Unloading became shorter in every group-by-jump cell (approximately 36–93 ms), and peak downward speed increased in every cell (approximately 0.033–0.160 m/s), with the smallest increase in high-level CMJ-A. Signal initiation was also associated with small differences in sagittal-plane joint angles, including estimated increases of 0.49° at the ankle, 1.81° at the knee, and 4.40° at the hip. These secondary kinematic findings complement the changes in countermovement depth and downward velocity, although their practical importance should be interpreted cautiously. Overall, the response extended across the full preparation-to-propulsion sequence rather than being confined to a single phase.

Estimates are signal-initiated minus self-initiated, averaged across groups and jump types. Models used all 564 valid attempts. Reported *p*-values are unadjusted; multiplicity corrections for the defined outcome groups are reported in the text. Because block order was fixed, these values are between-condition differences and do not isolate a causal signal effect.

### 3.4. Consistency Across Groups and Basketball-Specific Experience

Table 4 shows that overall group differences in the principal outcomes were modest. Across jump types and initiation conditions, the basketball groups did not differ clearly from controls in jump height (0.65 cm, 95% CI −1.76 to 3.07, *p* = 0.587), movement time (−0.032 s, 95% CI −0.080 to 0.017, *p* = 0.197), or RSImod (0.031 m/s, 95% CI −0.022 to 0.084, *p* = 0.251). High-level and mid-level players also did not differ clearly in jump height (1.82 cm, 95% CI −1.00 to 4.64, *p* = 0.199), movement time (0.004 s, 95% CI −0.053 to 0.061, *p* = 0.894), or RSImod (0.018 m/s, 95% CI −0.045 to 0.080, *p* = 0.572). This limited separation between groups is important when interpreting moderation as evidence of sport-specific expertise.

The main mechanical response to rapid initiation was broadly shared across groups. Basketball experience did not clearly moderate the condition effect for jump height (*p* = 0.092), movement time (*p* = 0.095), RSImod (*p* = 0.513), or propulsive net impulse (*p* = 0.390), and the high-versus-mid-level contrast was likewise unclear for these outcomes. The direction of the timing response was nevertheless consistent: movement time and unloading duration decreased in every group-by-jump cell, and peak downward speed increased in every cell. The data therefore support a common mechanical adjustment across groups more strongly than they support a systematic protective effect of basketball experience.

Several jump-type-specific group interactions were present, but they did not follow a monotonic expertise pattern. The experience-by-jump-type-by-condition interaction was 2.52 cm for jump height (95% CI 0.79 to 4.25, *p* = 0.005) and 0.081 N s/kg for propulsive net impulse (95% CI 0.023 to 0.139, *p* = 0.007). In CMJ-A; for example, the estimated change in jump height was −2.81 cm in controls, −0.45 cm in mid-level players, and −1.17 cm in high-level players—clearly not a linear progression with the competitive level. An isolated experience-by-condition interaction was also observed for peak downward speed (*p* = 0.032, unadjusted). Because these effects were exploratory, were not corrected as a single family, and did not form a consistent mechanical pattern, they do not support the conclusion that greater basketball expertise uniformly protects jump performance or mechanics.

### 3.5. Arm Swing and the Temporal Response

Arm swing changed the way participants responded to the added time constraint. The jump-type-by-condition interaction for movement time was 0.043 s (95% CI 0.019 to 0.068, *p* = 0.001), so the reduction in movement time was about 43 ms smaller in CMJ-A than in CMJ. Descriptively, movement time decreased by approximately 0.112 s in CMJ and 0.067 s in CMJ-A. The corresponding interaction for jump height was small and uncertain (0.29 cm, 95% CI −0.53 to 1.11, *p* = 0.479). Arm swing therefore clearly altered the timing response but did not clearly reduce the loss of jump height. The starting position of arms in CMJ-A should be taken into account as a possible limitation. The increase in RSImod was also smaller in CMJ-A (interaction −0.028 m/s, *p* = 0.024 before family-wise adjustment).

Several secondary jump-type-by-condition interactions pointed in the same direction. When arm swing was available, the reductions in unloading and braking duration were smaller, the change in countermovement depth was smaller, and the increase in mean propulsive force was attenuated. Because these secondary interactions were not uniformly robust after multiplicity control, they are best viewed as mechanical context for the main timing effect rather than as separate findings.

### 3.6. Reaction Time and Signal-to-Take-Off Time

The mean reaction time ranged from 260 to 285 ms across groups and jump types (Table 5). There was no clear difference between the basketball groups and controls (2.3 ms, 95% CI −22.3 to 27.0, *p* = 0.851), between high-level and mid-level players (17.0 ms, 95% CI −11.9 to 45.9, *p* = 0.242), or between CMJ-A and CMJ (6.2 ms, 95% CI −3.5 to 15.9, *p* = 0.201). The clear change in movement organization therefore occurred after movement onset, without evidence of a basketball-experience gradient in the initial reaction latency.

Total signal-to-take-off time was 0.100 s longer in CMJ-A than in CMJ (95% CI 0.085 to 0.114, *p* < 0.001). Neither basketball experience nor the high-versus-mid-level contrast showed a clear main effect. The longer total response time in CMJ-A was therefore explained mainly by the additional movement time after onset, rather than by a slower reaction to the signal.

Reaction time was also not clearly associated with jump height. After adjustment for movement time, a 100 ms longer within-participant reaction time corresponded to only about a 0.15 cm difference in jump height (95% CI −0.64 to 0.95, *p* = 0.700), and the between-participant estimate was similarly uncertain. Faster detection of the go-signal therefore did not explain which participants preserved jump height more successfully.

### 3.7. Sensitivity Analyses and Model Robustness

The principal condition effects were unchanged when the models were restricted to the first three valid attempts or to the three highest jumps in each cell. In the first-three analysis, the estimates were −1.61 cm for jump height, −0.091 s for movement time, and +0.033 m/s for RSImod. Using the three highest jumps produced corresponding estimates of −1.60 cm, −0.089 s, and +0.032 m/s (all *p* < 0.001). The main findings were therefore not sensitive to the trial-selection rule.

All 17 primary random-intercept mixed models converged, with participant intraclass correlation coefficients ranging from 0.24 to 0.91. Residual screening identified skewness or heavy tails in several secondary models, especially those for braking time and joint angles. Participant-clustered sensitivity analyses supported the three principal findings for jump height, movement time, and RSImod. The random-slope sensitivity models led to the same inference: the signal-initiated versus self-initiated contrast was −1.63 cm for jump height (95% CI −2.17 to −1.09), −0.089 s for movement time (95% CI −0.114 to −0.064), and +0.032 m/s for RSImod (95% CI 0.018 to 0.045; all *p* < 0.001).

## 4. Discussion

### 4.1. CMJ Organization Under the Rapid-Initiation Condition

The main finding is straightforward: differences in mechanical organization between the self-initiated and rapid-initiation conditions were more consistent than differences in final jump height. Movement time shortened in every group-by-jump cell, and the adjustment was visible from unloading through braking and propulsion. Participants used a faster, generally shallower countermovement and slightly higher mean propulsive force, but the shorter force-application time reduced net propulsive impulse and, to a lesser extent, jump height. This pattern was seen in physically active controls as well as mid-level and high-level basketball players. Arm swing changed how much the movement was compressed in time, whereas basketball-specific experience did not produce a clear or consistent gradient in the main condition effects [9,10,11,12,13,34]. These findings characterize performance during the complete visually triggered rapid-initiation task rather than the isolated effect of the visual signal.

### 4.2. Mechanical Strategy and Jump Height Are Related, but Not Equivalent

These results fit a broader pattern in the vertical-jump literature: jump height and the mechanical strategy used to produce it are related, but they are not the same outcome. Mandic and colleagues showed that experimentally changing countermovement depth can markedly alter force and power with only small effects on jump height [6]. They later found that athletes often choose a countermovement depth shallower than the depth that maximizes height, suggesting that the jump is not controlled solely around the height endpoint [7]. Markovic and colleagues reached a similar conclusion from a training perspective: different loading conditions produced comparable gains in jump height but distinct adaptations in CMJ depth, duration, force, and power [8]. Against that background, the present pattern is not surprising. The most consistent difference between the rapid-initiation and self-initiated conditions was in movement strategy, whereas the difference in jump height was smaller and more variable.

The mechanical sequence is also internally consistent. Participants spent less time in unloading and braking, moved downward faster, and generally used a shallower countermovement. Propulsion was then shorter as well. Mean net propulsive force was maintained or slightly increased, but the shorter application time reduced propulsive impulse. The small reduction in take-off velocity and jump height is therefore better interpreted as the cost of completing the movement sooner than as evidence of a generalized loss of force-producing capacity.

### 4.3. Mechanical Interpretation and Limits of Neurological Inference

The present findings should be interpreted at the level of external mechanics. In the rapid-initiation condition, participants completed the movement in less time, with shorter force–time phases, a generally shallower countermovement, and lower propulsive impulse. Studies of CMJs performed with an emphasis on quickness have reported a similar mechanical pattern, including shorter phase durations, reduced countermovement depth, and altered force and impulse characteristics [16,17]. Previous stimulus-triggered CMJ research has used electromyography to examine muscle-activation and response timing [19,20]; however, no EMG or other neurophysiological signals were recorded in the present study. Therefore, the observed mechanical differences cannot be attributed to changes in neural drive, muscle-activation timing, or any other neurological mechanism. RSImod further illustrates why a mechanical interpretation is more appropriate: it increased because movement time decreased proportionally more than jump height, not because absolute jump performance improved [32,33].

### 4.4. Arm Swing Changed the Available Mechanical Solution

Arm swing gave participants a different way to deal with the added time constraint. The reduction in movement time was about 43 ms smaller in CMJ-A than in CMJ, and the secondary preparation variables were broadly consistent with less compression when arm movement was allowed. This makes mechanical sense. Arm swing changes segmental coordination, can lengthen the countermovement, and contributes to vertical impulse and take-off velocity [9,10,11,34]. Its contribution is therefore not simply an increase in force; it also changes the coordination options available during the jump.

Arm swing did not, however, clearly preserve jump height across conditions. Signal-to-take-off time was about 100 ms longer in CMJ-A, while reaction time itself was similar between jump types, so the extra time occurred mainly after movement onset. The practical implication depends on the task: arm swing may allow a less compressed and more coordinated jump, but it also adds movement that must be completed before take-off.

### 4.5. Basketball-Specific Experience: No Simple Expertise Gradient

The three-group design allowed us to ask whether basketball-specific experience changed the response to rapid initiation. The clearest answer is that the core adjustment was shared. Movement time and unloading duration decreased across all groups, and the main group-by-condition interactions were unclear for jump height, movement time, RSImod, and propulsive impulse. Reaction time likewise showed no gradient with basketball experience. This is consistent with the task itself. Expertise advantages in reaction tests are usually specific to the information-response coupling being assessed [25,26,27,28,29], whereas a simple color change carries little basketball-specific information. Basketball experience would therefore not necessarily be expected to shorten the initial reaction latency.

The lack of a simple expertise gradient also agrees with earlier work on jump-control strategy. Mandic and colleagues found differences between elite basketball players and physically active non-athletes in preferred versus optimal countermovement depth, but not a straightforward quickness-height trade-off that could be attributed simply to basketball participation [7]. Here, several exploratory three-way interactions indicated that the groups differed for particular combinations of jump type and mechanical variable, but the pattern was not monotonic from control to mid-level to high-level. Because detailed training exposure (e.g., weekly training volume, resistance and plyometric training, competition frequency, and recent training history) was not recorded, and because the groups also differed in body size, these isolated differences cannot be attributed specifically to basketball experience. They may reflect other between-group differences or chance findings among multiple exploratory tests. Accordingly, the present data do not support the claim that high-level players are generally more resistant to the rapid-initiation requirement. The modest group separation in the principal CMJ outcomes further limits that interpretation [23,35].

### 4.6. Practical and Measurement Implications

A self-initiated CMJ and a maximal jump performed under a rapid-initiation requirement probe exhibited related, but not identical, capabilities. When the practical question is how quickly an athlete can organize and execute a maximal jump after an external event, jump height alone misses much of the response. Reaction time, movement time, signal-to-take-off time, countermovement depth and velocity, and propulsive impulse help show how the performance was produced. The size of the jump-height difference requires cautious interpretation. The mean reduction of 1.63 cm was approximately 4.4% of the self-initiated mean. Although its 95% confidence interval excluded zero, this demonstrates a group-average difference and does not establish a practically meaningful decline for an individual participant. Heishman et al. reported protocol-dependent smallest worthwhile changes in jump height of approximately 1.6–2.6 cm in NCAA Division I basketball players [11]. The present difference is therefore at the lower boundary of, or below, those benchmarks. Because those values were obtained in a different sample and no protocol-specific individual change threshold was established in the present study, they provide context rather than a definitive cutoff. Accordingly, the reduction in jump height should be considered small and of uncertain individual relevance, rather than evidence of a substantial impairment in jumping ability. Movement time, by contrast, shortened by approximately 91 ms (about 11% of the self-initiated value) and decreased in all six group-by-jump cells (range 45–124 ms), together with a coherent set of phase-level mechanical changes. The principal practical finding therefore concerns the altered timing and mechanical organization of the jump, not a large loss of jump height. The results also show why a higher RSImod should not automatically be read as better performance when the increase is driven mainly by a shorter denominator. More broadly, both testing and training studies should distinguish changes in mechanical strategy from changes in the final performance outcome [6,7,8].

### 4.7. Limitations

Several limitations should be considered. First, and most importantly, the fixed order of the experimental conditions is a major limitation. Within each jump type, the signal-initiated block always followed the corresponding self-initiated block. The conditions also differed in both the presence of a visual go-signal and the explicit instruction to initiate the jump as quickly as possible. Consequently, the independent contributions of the visual signal, the rapid-initiation instruction, and order-related effects, including learning, familiarization, and fatigue, cannot be separated. The randomized 3–10 s signal delay and catch trials were included to discourage temporal prediction and anticipatory movement initiation, but these procedures could not address confounding arising from the fixed block order. Accordingly, the observed contrasts should be interpreted as differences between the complete fixed-order task conditions rather than as isolated causal effects of the visual signal. Future studies should randomize or counterbalance both the initiation condition and arm-swing order. Second, the sample included only 44 men, with 14–15 participants per group. Although the groups were defined according to basketball participation and current competitive level, detailed training exposure was not quantified beyond the eligibility criteria. Session duration, weekly training volume, resistance or plyometric training, competition frequency, recent training history, and the physical activity volume of the non-basketball group were not systematically recorded. Consequently, the group comparisons reflect basketball participation and competitive classification rather than quantified differences in current training exposure. The relatively small baseline differences among groups also reduced sensitivity for detecting moderation by basketball experience and argue against treating the three categories as a simple linear expertise scale. Furthermore, the visual cue was a simple color change and did not contain basketball-specific perceptual information. The findings therefore concern whether basketball experience moderated a general visually triggered rapid-initiation task and should not be generalized to basketball-specific decision-making situations. Third, the number of valid attempts varied across participants, and several secondary outcomes had non-normal residuals. Trial-level mixed-effects models, adjustment for trial number, and the sensitivity analyses reduced these concerns but could not remove them completely. Finally, the present study assessed external mechanics only. Because EMG and other neurophysiological measures were not collected, the data cannot establish changes in neural drive, muscle-activation timing, or any other neurological mechanism.

## 5. Conclusions

When participants performed a maximal countermovement jump in response to an external go-signal under a rapid-initiation instruction, the movement was organized differently than during self-initiated jumping. The clearest adjustment was temporal: movement onset-to-take-off time shortened across all groups and both jump types, together with shorter preparation, a faster and generally shallower countermovement, shorter propulsion, and lower propulsive impulse. The accompanying mean reduction in jump height was small and should not be interpreted as a practically meaningful decrement for every individual. The principal finding concerns the altered temporal and mechanical organization of the jump rather than a substantial loss of jumping ability. Basketball-specific experience did not produce a clear overall gradient in this response, whereas arm swing consistently changed the timing solution. The findings therefore support interpreting maximal-jump performance as the product of an adaptable movement strategy, not as jump height alone. Because the visually triggered rapid-initiation condition always followed the self-initiated condition, the observed differences characterize performance under the complete fixed-order task condition and should not be attributed exclusively to the visual signal. These findings should be confirmed using a randomized or counterbalanced design.

## Figures and Tables

**Table 1:** Main outcomes, operational definitions, and units.

Role	Outcome	Operational Definition	Unit
Main performance	Jump height	Impulse-momentum estimate based on take-off velocity	cm
Main timing	Movement time	Unloading time + braking time + propulsion time	s
Combined measure	RSImod	Impulse-momentum jump height/movement time from movement onset to take-off	m/s
Signal-only timing	Reaction time	Visual signal to movement onset	s
Total response time	Signal-to-take-off time	Reaction time + movement time	s
Main mechanism	Net propulsive impulse	Net vertical-force impulse during propulsion/body mass	N s/kg
Impulse components	Impulse-equivalent mean net force	Normalized net propulsive impulse/propulsion time	N/kg
Impulse components	Propulsion duration	Lowest center-of-mass position to take-off	s
Preparation	Unloading duration	Movement onset to end of unloading	s
Preparation	Braking duration	Start of braking to zero vertical velocity	s
Preparation	Countermovement depth	Largest downward center-of-mass displacement/stature	fraction
Preparation	Peak downward speed	Largest downward vertical speed	m/s
Kinematics	Ankle, knee, and hip angles	Sagittal-plane joint angles at the lowest center-of-mass position	degrees

**Table 2:** Descriptive results for jump height, movement time, and RSImod by group, jump type, and initiation condition.

Group	Jump	Height Self-init. (cm)	Height Signal-Init. (cm)	Movement Self-Init. (s)	Movement Signal-Init. (s)	RSImod Self-Init. (m/s)	RSImod Signal-Init. (m/s)
Control	CMJ	34.24 ± 4.05	32.83 ± 3.65	0.821 ± 0.084	0.697 ± 0.100	0.423 ± 0.074	0.481 ± 0.085
Mid-level	CMJ	33.83 ± 3.27	32.29 ± 4.28	0.770 ± 0.118	0.670 ± 0.079	0.453 ± 0.102	0.492 ± 0.109
High-level	CMJ	35.65 ± 3.70	33.66 ± 3.36	0.781 ± 0.103	0.667 ± 0.065	0.467 ± 0.085	0.511 ± 0.084
Control	CMJ-A	40.16 ± 4.76	37.18 ± 3.52	0.863 ± 0.088	0.782 ± 0.095	0.475 ± 0.087	0.482 ± 0.072
Mid-level	CMJ-A	38.56 ± 5.17	38.04 ± 5.14	0.831 ± 0.114	0.755 ± 0.076	0.480 ± 0.111	0.512 ± 0.105
High-level	CMJ-A	41.13 ± 3.61	39.87 ± 3.95	0.821 ± 0.089	0.781 ± 0.066	0.513 ± 0.075	0.518 ± 0.084

Values are mean ± SD of participant-level condition means. Control, n = 15; mid-level, n = 15; high-level, n = 14.

**Table 3:** Model-estimated changes under the rapid-initiation requirement, averaged across groups and jump types.

Outcome	Estimate	95% CI	*p*	Unit
Jump height	−1.63	−2.04 to −1.21	<0.001	cm
Movement time	−0.091	−0.103 to −0.078	<0.001	s
RSImod	0.032	0.023 to 0.041	<0.001	m/s
Net propulsive impulse	−0.052	−0.066 to −0.039	<0.001	N s/kg
Mean net propulsive force	0.274	0.133 to 0.415	<0.001	N/kg
Propulsion time	−0.010	−0.013 to −0.008	<0.001	s
Unloading time	−0.069	−0.079 to −0.060	<0.001	s
Braking time	−0.011	−0.017 to −0.005	<0.001	s
Depth/stature	−0.0058	−0.0090 to −0.0026	<0.001	fraction
Peak downward speed	0.103	0.080 to 0.125	<0.001	m/s
Ankle angle	0.49	0.12 to 0.86	0.010	degrees
Knee angle	1.81	0.97 to 2.64	<0.001	degrees
Hip angle	4.40	3.03 to 5.76	<0.001	degrees

**Table 4:** Model-estimated jump-height changes under the rapid-initiation requirement by group and jump type.

Group	Jump	Estimate (cm)	95% CI	*p*
Control	CMJ	−1.43	−2.42 to −0.44	0.006
Control	CMJ-A	−2.81	−3.81 to −1.82	<0.001
Mid-level	CMJ	−1.79	−2.81 to −0.78	0.001
Mid-level	CMJ-A	−0.45	−1.45 to 0.55	0.368
High-level	CMJ	−2.09	−3.12 to −1.06	<0.001
High-level	CMJ-A	−1.17	−2.18 to −0.16	0.024

Estimates are signal-initiated minus self-initiated. The cell estimates illustrate the exploratory group-by-jump-type interaction and should not be interpreted as a linear expertise gradient.

**Table 5:** Reaction time and total signal-to-take-off time in the signal-initiated blocks.

Group	Jump	Reaction Time (ms)	Signal-to-Take-Off Time (s)
Control	CMJ	272 ± 41	0.969 ± 0.119
Mid-level	CMJ	260 ± 31	0.931 ± 0.074
High-level	CMJ	283 ± 51	0.950 ± 0.087
Control	CMJ-A	275 ± 43	1.057 ± 0.125
Mid-level	CMJ-A	274 ± 47	1.028 ± 0.087
High-level	CMJ-A	285 ± 47	1.066 ± 0.087

Values are mean ± SD of participant-level means from the signal-initiated blocks.

## Data Availability

The data presented in this study are available from the corresponding author on reasonable request. The data are not publicly available because of restrictions related to participant privacy.
